# Predictors of quality of life: A quantitative investigation of the stress-coping model in children with asthma

**DOI:** 10.1186/1477-7525-6-24

**Published:** 2008-03-26

**Authors:** Yvette Peeters, Sandra N Boersma, Hendrik M Koopman

**Affiliations:** 1 Medical Decision Making, University Medical Centre Leiden, PO Box 9600, 2300 RC Leiden, The Netherlands; 2 Medical Psychology, University Medical Centre Leiden, PO Box 9555, 2300 RB Leiden, The Netherlands

## Abstract

**Background:**

Aim of this study is to further explore predictors of health related quality of life in children with asthma using factors derived from to the extended stress-coping model. While the stress-coping model has often been used as a frame of reference in studying health related quality of life in chronic illness, few have actually tested the model in children with asthma.

**Method:**

In this survey study data were obtained by means of self-report questionnaires from seventy-eight children with asthma and their parents. Based on data derived from these questionnaires the constructs of the extended stress-coping model were assessed, using regression analysis and path analysis.

**Results:**

The results of both regression analysis and path analysis reveal tentative support for the proposed relationships between predictors and health related quality of life in the stress-coping model. Moreover, as indicated in the stress-coping model, HRQoL is only directly predicted by coping. Both coping strategies 'emotional reaction' (significantly) and 'avoidance' are directly related to HRQoL.

**Conclusion:**

In children with asthma, the extended stress-coping model appears to be a useful theoretical framework for understanding the impact of the illness on their quality of life. Consequently, the factors suggested by this model should be taken into account when designing optimal psychosocial-care interventions.

## Background

Children with asthma have a lower health related quality of life (HRQoL) than healthy children [1-3] and children with severe asthma report an even lower HRQoL compared to children with mild asthma [2]. Quality of life has been defined as the individuals' perception of their position in life in the context of the culture and value systems in which they live, in relation to their goals, expectations, standards and concerns [4]. Naturally *health related *quality of life stands for the quality of life in relation to one's health. A better understanding of the different aspects of, and influences on HRQoL is necessary to be able to offer optimal psychosocial-care to children with asthma.

Stress and negative emotions of a chronic illness such as asthma often result in anxiety, depression and anger which affect HRQoL [5]. At the same time, coping-style appears to be an important psychosocial moderator between stress and negative emotions on the one hand, and HRQoL on the other hand [6]. Individual differences in coping with a chronic illness are described by several theories of stress and emotion [7]. One of these theories is the cognitive-appraisal model of Lazarus and Folkman [8]. With their theory they show that a person confronted with a stressor firstly evaluates this stressor and secondly determines his or her emotional or behavioural reaction. That is, the person evaluates whether there is potential harm or benefit (primary appraisal) and consequently decides what can be done to deal with the situation (secondary appraisal). An event is appraised as stressful when primary appraisals exceed secondary appraisals, and by using coping processes a person might be able to reduce this stress [8].

**Figure 1:**
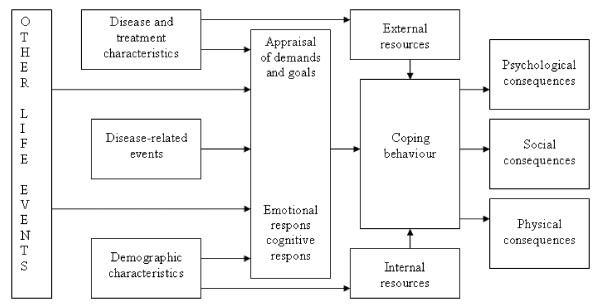
Stress-coping model of Maes, Leventhal & De Ridder (1996).

Derived from this cognitive-appraisal model an extended model for coping with a chronic disease was developed by Maes, Leventhal and de Ridder [5]. Figure 1 shows this extended stress-coping model. Based on the model, other life events, disease characteristics, disease-related events, and demographic characteristics are linked to the appraisal of demands and goals. Furthermore, all factors are directly or indirectly related to coping behaviour, which itself is also moderated by external – and internal resources. Finally, all these factors together contribute to psychological, social and physical consequences (HRQoL) trough coping.

To our knowledge only a few studies investigated the extended stress-coping model in total [9-11]. In a sample of adult heart patients, tentative proof was found for the model in total [9,10]. Moreover, Röder et al. [11] investigated this stress-coping model in children with asthma in a school context. In their study three different disease-related stressors were included: problems with school work, shortness of breath and rejection by peers. It appeared that all three stressors contributed to the explanation of differences found in psychosocial functioning. However, interrelations between the different factors in the prediction of HRQoL remained unclear.

The aim of our study is to further explore predictors of HRQoL with the stress-coping model resulting towards a better understanding of HRQoL of children with asthma in a general context.

## Method

### Participants and procedure

The sample for this study was obtained from the European DISABility KIDS (DISABKIDS) project [12]. In the DISABKIDS project, children with a chronic illness and their parents were asked to participate in the study, while they visited a paediatric hospital. After informed consent was obtained, children and their parents were asked to fill in self-reported questionnaires, which were handed out or mailed [13]. After a 2–4 weeks interval parents and children were asked to fill in another questionnaire. This questionnaire was similar to the first except for an additional coping questionnaire and without questions about demographic characteristics. For the present study, only the children with asthma from Austria, Germany, Sweden and The Netherlands were selected since in these countries the same HRQoL questionnaire was elected during re-test. The ethics review committee of the different paediatric hospitals approved the research protocol.

### Instruments and measures

The constructs of the stress-coping model were assessed with a selection of all questionnaires developed specifically for the DISABKIDS project [14]. For 'demographic characteristics', age of the child at time of participation, educational level of the parents and living environment were selected. 'Education of the parents' reflects the highest completed education of the parent who filled out the questionnaire. 'Living environment' was rated by the parent according to three categories: village, small town or big city.

In the questionnaires administered in the DISABKIDS asthma module four additional questions answered by the parent about the treatment of their child's asthma like "Did your child visit a specialist in the last 12 months?" were asked. To assess 'treatment characteristics' an index was created based on these four questions. In addition, children were asked whether they use medicine or not.

Based on questionnaires only answered by the parents, 'asthma severity', 'social support' and 'internal resources' were assessed. Since asthma is subject to change on a daily basis there is no standard way to score the severity of asthma [14]. However, in the DISABKIDS project 'asthma severity' was based on the scale of Rosier et al. [15], a questionnaire consisting of six questions answered by a four point Likert scale, ranging from 0 (never) to 4 (daily). The score on this questionnaire was categorised in low, mild, moderate or severe asthma. 'Social support' was measured by the single item, single or two parent families and by three questions about the accessibility of social support. Scores on the questions about accessibility of social support were weighted and totalled. Finally to indicate 'internal resources', overall development on a 3 point likert-scale, and occurrence of physical, emotional or social problems of the child was rated. Based four questions answered by the parent about the treatment of their child's asthma like "Did your child visit a specialist in the last 12 months?" on the answers an index for 'internal resources' was created.

**Figure 2:**
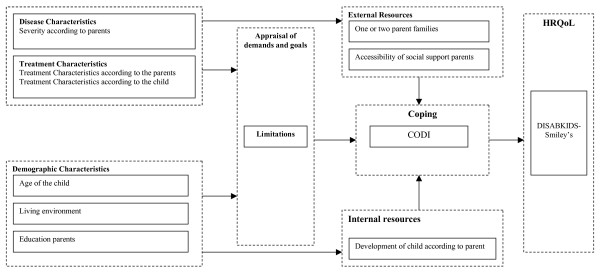
Stress-coping model with Instruments and Measures.

Some questionnaires only answered by the children, were used to assess appraisal of demands and goals, coping behaviour and HRQoL. To assess 'appraisal of demands and goals' the domain limitations from the DISABKIDS Chronic Generic Measure (DCGM-37) [14] was used. If children experience limitations they will have difficulties with a particular event and with pursuing their goals. 'Coping' was assessed with the COping with a DIsease (CODI) questionnaire [16], a coping questionnaire which includes six coping strategies: acceptance, avoidance, cognitive-palliative, distance, emotional reaction and wishful thinking. Finally the 12 item DISABKIDS-Smiley's [14] was answered to assess HQoL. This scale is associated with other measures of HRQoL like, the revised children quality of life questionnaire (KINDL) [17], and discriminates between different levels of clinical severity [14]. In Figure 2 an overview is given of which indicators were used to assess the different constructs of the stress-coping model.

### Data analysis

**Figure 3:**
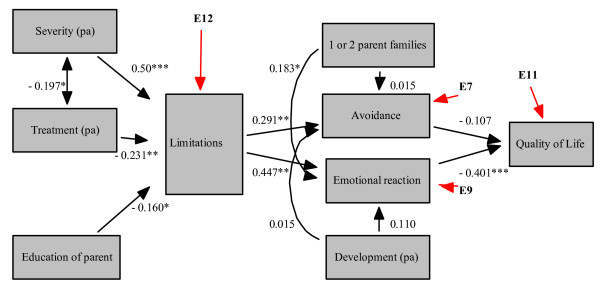
**Path model with standardized path coefficients**. Significance of the parameter estimates: *p < 0.10, **p < 0.05, ***p < 0.01, pa stands for answer given by the parent

Prior to the analyses, all variables were examined for multi- and univariate outliers, missing values, normality, and linearity. Missing data were excluded list-wise. Pearson correlations were used to examine the associations between the variables, followed by different regression analyses to explore possible multivariate associations. A path analysis was conducted to test the fit of our data on the specified model (Figure 3). A good fit is indicated by a non-significant chi squared statistic, a Comparative Fit Index [CFI] < 1.0, a Bentler-Bonett Non-normed fit index [NNFI] < 0.95 and by a Root mean-square error of approximation [RMSEA] < 0.05.

### Results

From the 280 eligible children, 193 were not included in the re-test. The re-test was conducted by the DISABKIDS project to investigate the reliability of their questionnaires. However only in this re-test a coping questionnaire was added. For this reason exclusively data from children selected in the re-test could be included. The final sample of this study comprised 34 girls and 53 boys, between 7 and 16 years old. As judged by their parents, 5 children had severe, 15 moderate, 19 mild and 45 low severe asthma; data of 3 children were missing. Sixty-nine (79%) of all questionnaires answered by a parent was answered by the mother.

One univariate outlier and four cases with missing values on more then three variables were identified and removed. Normal distributions of and linearity between all variables in the study were found to be satisfactory except for one of the subscales of the CODI the variable 'wishful thinking' (Skewness = -1.574 with *SD*. = 0.257; Kurtosis = 1.909 with *SD*. = 0.508). This variable was therefore not included in further analyses.

All instruments revealed a satisfactory reliability with Cronbachs' alphas between 0.69 and 0.84. As a guideline, Cronbach alpha values of 0.7 are regarded as satisfactory applying scales to further analysis [18]. The instrument used to measure 'treatment characteristics', including four questions about the treatment of the child answered by the parent, revealed a relatively low Cronbachs' alpha. However, the items in this questionnaire are not necessarily related to each other. That is, asthma treated with medication prescribed by a physician is not necessary related to self medication. In the construction of the DISABKIDS instruments three age groups were used, 4–7 years, 8–12 years and 13–16 years [16]. Yet, in this study 'age' was dichotomised into younger and older than 12 years, since only one child was younger than 8 years.

#### Univariate relationships

Table 1 shows the Pearson correlation coefficients and levels of significance, of possible correlates of 'appraisal of demands and goals' which is represented by the variable 'limitations'.

**Table 1:** Pearson correlations (n) between predictors and limitations.

	Limitations
Severity (parent)	.510** (81)
Treatment (parent)	-.307** (78)
Treatment (child)	-.285* (80)
Education of Parent	-.151 (80)
Living Environment	-.134 (80)
Age	-.023 (81)

* *p *< .05, ** *p *< .01

Table 2 shows the Pearson correlation coefficients between possible correlates of and the six coping scales. Only 'Limitations', was significantly correlated with all coping scales except for 'cognitive-palliative coping'. The variable 'development of the child' was significantly related to the coping scale 'emotional reaction'.

**Table 2:** Pearson correlations (n) between predictors and six coping scales

	Avoiding	Acceptance	Cognitive-palliative	Distance	Emotional reaction
Limitations	.256* (78)	-.479* (77)	.195 (78)	-.303* (79)	.480** (78)
Single or two parent family	-.002 (79)	.017 (78)	-.130 (79)	.097 (80)	.159 (79)
Accessibility of social support	-.127 (76)	.142 (75)	-.053 (76)	.111 (77)	-.113 (76)
Development child (parent)	.128 (76)	-.156 (75)	.222 (76)	.079 (77)	.271* (76)

* *p *< .05, ** *p *< .01

Table 3 shows that only the coping scales 'acceptance' and 'emotional reaction' were significantly correlated with HRQoL.

**Table 3:** Pearson correlations (n) between coping scales and quality of life

	Quality of Life
Avoiding	.194 (78)
Acceptance	.427** (77)
Cognitive-palliative	-.083 (78)
Distance	.101 (79)
Emotional reaction	-.412**(78)

* *p *< .05, ** *p *< .01

#### Testing the model

Since there were too many variables compared to our sample size, to test the model, a selection of the variables had to be made. Treatment variables answered by the parents appeared to have a slightly higher correlation with limitations than those answered by the children. Moreover, in general variables answered by parents tend to be more reliable [19]. In further analyses answers given by the parents were therefore used to assess 'treatment characteristics'. With respect to the different coping variables, no more than two variables could be included. Both the variable 'avoidance' and 'emotional reaction' were selected; the variable 'acceptance' was excluded due to a significant multivariate kurtosis.

The model that follows from this selection, with three demographic variables and two external resources, showed a poor fit. Including the demographic variables and 'external resources' one by one, however, revealed a model with a good fit (Chi square (24,70) = 28.012, *p *= 0.259; Comparative Fit Index [CFI] = 0.943; Bentler-Bonett Non-normed fit index [NNFI] = 0.914; Root mean-square error of approximation [RMSEA] = 0.049). Within this model, 'age of the child', 'living environment' and 'accessibility of social support by the parents' were excluded. Correlations of the other demographic variables and external resources can be seen in, respectively, table 1 and 2.

#### Regression analyses

Table 4 shows the results of four separate regression analyses. The first regression analysis showed that children with severe asthma experienced more limitations than children with moderate or low severe asthma. In addition, children who received more treatment, experienced less limitations. No significant associations between 'limitations' and education of the parents were found.

**Table 4:** Regression analyses

Independent Variable	β	*B*	*T*
Dependent variable: Limitations			
Severity of disease (pa^1^)	.451	.378	4.491**
Treatment (pa^1^)	-.210	-.931	-2.080*
Education of the parent	-.140	-.068	-1.418
*R *square = 0.310, *F*(3,76) = 10.934, *p *< 0.001			
Dependent variable: Avoidance			
Limitations	.259	.318	2.137*
single ore two parent family	-.160	-.042	-0.137
Development of child (pa^1^)	.024	.063	0.198
*R *square = 0.072, *F *(3,74) = 1.837, ns			
Dependent variable: Emotional Reaction			
Limitations	.446	.353	4.168**
single ore two parent family	.156	.268	1.538
Development of child (pa^1^)	.115	.193	1.067
*R *square = 0.275, *F*(3,74) = 8.991, *p *< 0.001			
Dependent variable: Quality of Life			
Avoidance	-.093	-.044	-0.856
Emotional Reaction	-.387	-.286	-3.572**
*R *square = 0.178, *F*(2,77) = 8.094, *p *= 0.001			

*p *< .05, ** *p *< .01

^1^pa stands for answer given by the parent

In the regression analysis with 'avoidance' as dependent variable, only 'limitations' showed a significant relationship with avoidance with no more than 7% of the variance explained. With regard to 'emotional reaction', it can be seen that a child who experienced more limitations more often reported to use emotional reaction as a coping strategy. Finally children, who reported to use mostly emotional reaction as a coping strategy, reported a lower quality of life.

#### Path analysis

Figure 3 presents the model analysed with path analysis, which appeared to have a good fit; Chi square (24,70) = 28.012, *p *= 0.259; Comparative Fit Index [CFI] = 0.943; Bentler-Bonett Non-normed fit index [NNFI] = 0.914; Root mean-square error of approximation [RMSEA] = 0.049.

The parameter estimates showed the same pattern as in the regression analyses. 'Limitations' was best predicted by 'severity of the disease' and 'treatment'. Again, the relationship between 'treatment' and 'limitations' was negative.

'Limitations' was the only significant predictor of 'avoidance', whereas 'emotional reaction' was predicted by both 'limitations' and 'one or two parent families'. Moreover, 'emotional reaction' was the only significant predictor of quality of life.

At last several post-hoc analyses were conducted in order to examine if the data might fit another model even better, compared to the extended stress-coping model. The Multivariate Lagrange Multiplier Test showed only a significant relation between 'development of the child' and 'limitations' with a standardized parameter of 0.257 (*p *< 0.05). Finally, the Wald test indicated that no observed associations could be removed from the model.

## Discussion

The aim of the present study was to further explore predictors of HRQoL of children with asthma. This study is, to our knowledge, the first study that investigated predictors of HRQoL in children with asthma within the context of other predictors. With this study a first step is made in investigating predictors of HRQoL while taking other predictors in account. Since most studies investigate only direct relations between predictors and HRQoL it is difficult to compare results of these studies to the results found in the present study.

In the present study, tentative support was found for the notion that the stress-coping model reflects most of the relationships between the included predictors and HRQoL for children with asthma. Besides coping, no other predictors appeared to have a direct relation with HRQoL. In contrast to our results, Röder et al. [11] found more direct predictors of HRQoL besides coping. Yet, in their study concepts and variables were independently investigated and a restriction was made by investigating factors related to a school context. Several other studies also found direct predictors of HRQoL such as severity of asthma [1-3,20]. However the problem is that they also concentrated only on the direct relations between HRQoL and severity of asthma.

Several non-significant associations were found in the model. Non-significant associations imply that the parameters do not differ from zero and could be deleted from the model. However, such a decision should be based primarily on theoretical considerations [21]. Since Maes et al. [5] postulated that internal and external resources have a relationship with coping, it was decided to keep the non-significant associations in the model.

In the present study, most support was found for the axis of the stress-coping model. Disease characteristics, appraisal of the disease, coping, and quality of life are all significantly related to each other. This part of the extended stress-coping model might be seen as a representation of the theory of Lazarus and Folkman [8], which indicates that this study possibly reveals some tentative support for this theory.

Furthermore, avoidance coping strategy of the child had only little influence on HRQoL. This finding seems to confirm Hesselink et al. [22] who found that an avoidance coping strategy was important for predicting quality of life for adult patients with asthma. However, by including emotional reaction as coping style in their study, the effect of avoidance disappeared as well [22]. In the present study, we included both avoidance and emotional reaction as coping strategies. Possibly, the association between emotional reaction and quality of life is that strong, that it obscured the association between avoidance and quality of life.

For patients with chronic obstructive pulmonary disease (COPD) perceptions of personal control was related to better HRQoL [23]. It might be possible that in the present study children with a tendency to use emotional reaction as coping strategy felt that they had less control over their disease, which might have lead to a worse HRQoL.

Finally, the finding that children with more treatment experience fewer limitations might possibly be explained by undertreatment. Both physician under-prescription of inhaled corticosteroids and the underuse by children is associated with higher hospitalisation rates and less asthma control [24]. It is known that children with asthma do not use their inhaled corticosteroids as often as they should [25].

The points described above should be made with caution due to some methodological limitations. First, the questionnaires used in this study were not originally created to specifically measure the constructs of the stress-coping model. In future studies it would be desirable to put effort in developing variables specially for predicting the factors as described by Maes et al. [5]. Indicating factors with more than one predictor would be desirable as well.

Secondly, some of the aspects in the model had to be left out. In regard to the small sample size, too many parameters had to be estimated when all associations of the extended stress-coping model were included [26]. In future studies a larger sample size is needed to investigate all aspects of the extended stress-coping model. Despite the exclusion of some of the aspects of the model, the power to detect a model without a good fit remained small; < 0.20 [27]. As a consequence, the low values of the test statistics might either reflect correctness of our model or lack of sensitivity to error [21]. On the other hand, the similarity of results with regression analyses gives some support to the results from the path-analysis.

Furthermore, the children recruited are from 4 different countries, so that the results might be affected by cultural or language differences. Data from the parents were received from the mothers in 69 of the 87 cases. It could be expected that answers given by fathers are different. However in this study no difference were found between answers given by the parents except for education, compared to the fathers, mothers were higher educated.

It would be worthwhile to explore whether similar results hold for children followed several years. Lanfolt et al [28] found that HRQoL significantly increased over a year. However their study focussed on children diagnosed with cancer.

The findings described in this study are specific for children with asthma, it remains uncertain how this generalises to other patient groups. For example, the finding that emotional reaction as coping strategy negatively influences HRQoL might turn out to be positive for other patient groups. The efficacy of a particular coping strategy is likely to depend on the nature of the stressful situation. Emotion-focused coping are associated with lower levels of distress in situations that are not controllable [29]. Since asthma is controllable with effective management [30] emotion focussed coping might have a negative influence on HRQoL. Furthermore this study was conducted by children between seven and 12 years old. For adolescents adherence to prescribed medication and attack management is low [31]. This difference in adherence between age groups might quite possible have influence on the relation between severity of the disease, treatment and the limitations that one experience.

Regarding to the coping strategy, in this study relation was found between one or two parent families and the use of emotional reaction as coping strategy. Most likely for adult patients the impact of their growing up in one or two parent families is smaller than for children living in this situation.

## Conclusion

The results of this study gave tentative support for the notion that the stress-coping model reflects most of the relationships between the included predictors and HRQoL for children with asthma. A first step is made in identifying predictors of quality of life for children with asthma. However, future research is necessary to analyse the model and with that predictors of quality of life further. Although our results are preliminary, it seems that the factors suggested by this model are important and should be taken into account when designing optimal psychosocial-care interventions.

## Abbreviations

HRQoL Health Related Quality of Life

DISABKIDS DISABility KIDS project

CODI COping with a DIsease

KINDL the revised children quality of life questionnaire

## Competing interests

The author(s) declare that they have no competing interests.

## Authors' contributions

HMK developed the core idea and was involved with the DISABKIDS project which made it possible to use the data from this project. SB and YP conducted the literature search. YP performed the statistical analyses; all authors were involved in the interpretation of the results. YP wrote the first draft of the paper. All authors revised the first draft critically and gave final approval of the version published.
